# Pediatric Invasive Pneumococcal Disease Three Years after PCV13 Introduction in the National Immunization Plan—The Continued Importance of Serotype 3

**DOI:** 10.3390/microorganisms9071428

**Published:** 2021-07-01

**Authors:** Catarina Silva-Costa, Joana Gomes-Silva, Lúcia Prados, Mário Ramirez, José Melo-Cristino, on behalf of the Portuguese Group for the Study of Streptococcal Infections, the Portuguese Study Group of Invasive Pneumococcal Disease of the Pediatric Infectious Disease Society

**Affiliations:** Faculdade de Medicina, Instituto de Microbiologia, Instituto de Medicina Molecular, Universidade de Lisboa, 1649-028 Lisboa, Portugal; anacosta@fm.ul.pt (C.S.-C.); jfsilva@medicina.ulisboa.pt (J.G.-S.); luciaprados@medicina.ulisboa.pt (L.P.); melo_cristino@fm.ul.pt (J.M.-C.)

**Keywords:** conjugate vaccine, serotype, invasive disease, epidemiology, molecular diagnostics, antimicrobial resistance

## Abstract

The introduction of pneumococcal conjugate vaccines PCV7 and PCV13 led to decreases in incidence of pediatric invasive pneumococcal disease (pIPD) and changes in serotype distribution. We evaluated the consequences of higher vaccine uptake after the introduction of PCV13 in the National Immunization Plan (NIP) in 2015. Besides culture and conventional serotyping, the use of molecular methods to detect and serotype pneumococci in both pleural and cerebrospinal fluid samples contributed to 30% of all pIPD (*n* = 232) in 2015–2018. The most frequently detected serotypes were: 3 (*n* = 59, 26%), 10A (*n* = 17, 8%), 8 (*n* = 16, 7%) and 19A (*n* = 10, 4%). PCV13 serotypes still accounted for 46% of pIPD cases. Serotypes not included in any currently available conjugate vaccine (NVT) are becoming important causes of pIPD, with the increases in serotypes 8 and 33F being of particular concern given the importance of serotype 8 in adult IPD and the antimicrobial resistance of serotype 33F isolates. This study highlights the importance of using molecular methods in pIPD surveillance since these allowed a better case ascertainment and the identification of serotype 3 as the leading cause of pIPD. Even in a situation of vaccine uptake >95% for 3 years, PCV13 serotypes remain important causes of pIPD.

## 1. Introduction

Pneumococcal conjugate vaccines (PCVs) have already proven to be highly effective in preventing invasive pneumococcal disease (IPD). Routine infant immunization led to an overall decrease in the incidence of pediatric IPD (pIPD), but also indirectly in non-immunized adults through the reduction of carriage and transmission of vaccine serotypes [1,2,3,4,5,6,7,8]. Additionally, changes in serotype distribution were also detected, with a decrease in the proportion of IPD cases caused by serotypes included in vaccine formulations, with the notable exception of serotype 3, which has not been decreasing as much in most countries where PCV13 is being used [8,9,10,11].

In Portugal, the introduction of conjugate vaccines began in 2001, when PCV7, targeting serotypes 4, 6B, 9V, 14, 18C, 19F and 23F, became available. Soon after this, the proportion of pediatric IPD cases caused by serotypes included in the vaccine decreased, mostly due to declines of serotypes 4, 6B, 14 and 23F [2]. However, similar to many other countries, these changes were accompanied by a rise in non-PCV7 serotypes 1, 7F and 19A [1,2]. Higher valency vaccines became available in 2009 (PCV10, targeting PCV7 serotypes and serotypes 1, 5 and 7F) and in 2010 (PCV13, targeting PCV10 serotype and serotypes 3, 6A and 19A). Even though only available through the private market, PCV7 uptake was high, reaching 75% around 2008 [1] but, after PCV13 introduction, vaccine uptake decreased to 62% and remained approximately constant until 2014 [12]. Besides the changes in serotype distribution, it was also possible to detect a decrease in the incidence of pIPD from 8.19 cases per 100,000 in 2008–2009 to 4.52/100,000 in 2011–2012. In contrast, from 2012–2013 to 2014–2015, the incidence of pIPD varied only slightly and without a clear trend, suggesting that no further benefits of vaccination could be expected with the coverage existing at the time. Moreover, PCV13 serotypes were still responsible for the majority of pIPD cases (58%), of which serotypes 3, 14 and 1 were the most frequently detected. The frequent detection of serotype 3, only possible due to the use of molecular methods, such as real-time PCR, was particularly worrisome because it was associated with vaccine failures in a considerable number of cases [3,12]. Potential reasons for the persistence of PCV serotypes could include a high antimicrobial consumption and the resistance found in some of the PCV serotypes, particularly isolates of serotypes 14, 19A, 19F and 6B [3], a slower vaccine uptake or a lower vaccination coverage when compared to countries where PCVs are included in the national immunization program (NIP). At the same time the non-PCV13 serotypes 15B/C, 24F and 10A became important causes of disease [3], in agreement with what was being reported in other European countries [13].

In July 2015, PCV13 was introduced in the NIP for children born after January 2015, in a 2 + 1 schedule, with doses given at 2, 4 and 12 months of age, quickly reaching >95% coverage [14]. Two other higher valency vaccines, a 15-valent (PCV15) and a 20-valent (PCV20) vaccine are currently obtaining regulatory approval [15,16] potentially offering additional prevention options for pIPD. The aims of this work were to determine the serotype distribution and antimicrobial susceptibility profiles of the pneumococcal population causing pIPD in a period of almost universal vaccination with PCV13.

## 2. Materials and Methods

The Portuguese Group for the Study of Streptococcal Infections and the Portuguese Study Group of Invasive Pneumococcal Disease of the Pediatric Infectious Disease Society have been monitoring pneumococcal invasive infections in Portugal since 2007. The study was approved by the Institutional Review Board of the Centro Académico de Medicina de Lisboa. As these were considered surveillance activities, they were exempt from informed consent. All methods were performed in accordance with the relevant guidelines and regulations.

The data and isolates were de-identified so that these were irretrievably unlinked to an identifiable person. Reporting of isolates and patient samples was performed by the microbiology laboratories and pediatric departments of 55 hospitals throughout Portugal. All centers reported during the entire period. A case of pIPD was defined as a person (<18 years) from whom an isolate of *S. pneumoniae* was recovered from a normally sterile body site (not including middle ear fluid) or from whom pneumococcal DNA was detected in cerebrospinal fluid (CSF) or pleural fluid. Only isolates recovered between 1 July 2015 and 30 June 2018 were included in the present study. Epidemiological years were defined as spanning from 1 July to 30 June of the following year. Only one isolate from each patient in a 90-day interval was included. Cases ascertained and isolates and samples recovered up to June 2015 were previously characterized [1,2,3,17,18]. Strains were identified as *S. pneumoniae* by colony morphology and hemolysis on blood agar plates, optochin susceptibility and bile solubility. In the case where the IPD diagnosis was made by molecular methods, the detection of pneumococci was performed by molecular methods using two *S. pneumoniae* genes (*lytA* and *wzg*) used for identification [12].

Serotyping was performed by the standard capsular reaction test using the chessboard system and specific sera (Statens Serum Institut, Copenhagen, Denmark). Given the high frequency of spontaneous switching between serotypes 15B and 15C, we decided to group isolates with these serotypes into a single group. Additionally, given the difficulties in phenotypically distinguishing isolates of serotypes 25A and 38, as well as isolates with serotypes 29 and 35B, these also grouped together into 25A/38 and 29/35B, respectively. In cases where the diagnosis was performed by molecular methods, the serotypes were also determined by real-time PCR with a reaction targeting 21 serotypes [12]. Serotypes were classified into vaccine serotypes (VT), i.e., those included in PCV7 (serotypes 4, 6B, 9V, 14, 18C, 19F, 23F), the additional three found in PCV10 relative to PCV7 (addPCV10: 1, 5, 7F), the additional three found in PCV13 relative to PCV10 (addPCV13: 3, 6A, 19A), and non-vaccine serotypes (NVT) indicating serotypes not included in PCV13. The additional two serotypes included in PCV15 relative to PCV13 (addPCV15: 22F, 33F) and the additional five serotypes included in PCV20 relative to PCV15 (addPCV20: 8, 10A, 11A, 12F, 15B) were also discriminated. Since the PCR reaction includes probes for all PCV13 serotypes, when a serotype could not be identified using this method the case was grouped into the NVT cases. The only serotype included in PCV20 and not detected by the PCR reaction employed is serotype 10A but other serotypes could also not be distinguished from other closely related serotypes [12] and are indicated as such. Etest strips (AB Biodisk, Solna, Sweden) were used to determine the minimal inhibitory concentrations (MICs) for penicillin, cefotaxime, ceftriaxone, and levofloxacin. Unless otherwise stated, we used the CLSI-recommended breakpoints for oral penicillin [19] as epidemiological breakpoints, allowing the comparison with previous studies.

Isolates were further characterized by determining their susceptibility to erythromycin, clindamycin, vancomycin, linezolid, tetracycline, trimethoprim–sulfamethoxazole and chloramphenicol by the Kirby–Bauer disk diffusion technique, according to the CLSI recommendations and interpretative criteria [19]. Macrolide resistance phenotypes were identified using a double-disc test with erythromycin and clindamycin. Simultaneous resistance to erythromycin and clindamycin defines the MLS_B_ phenotype (resistance to macrolides, lincosamides and streptogramin B) while non-susceptibility only to erythromycin indicates the M phenotype.

The Fisher exact test was used for differences in proportion with the false discovery rate (FDR) correction for multiple testing [20], and the Cochran–Armitage test was used for trends. A *p* < 0.05 was considered significant for all tests.

## 3. Results

Between July 2015 and June 2018, 223 IPD cases were reported. In 70 cases (31.4%) diagnosis was only possible using molecular methods directly from patient samples and in the remaining 153 cases (68.6%) an isolate was sent to our laboratory for characterization. Most available isolates were recovered from blood (*n* = 115, 75.5%), with the remaining being recovered from CSF (*n* = 24, 15.7%), pleural fluid (*n* = 8, 5.2%), peritoneal fluid (*n* = 5, 3.3%) and synovial fluid (*n* = 1, 0.6%). Among the 70 samples for which only pneumococcal DNA was available, pleural fluid was the most frequent patient sample (*n* = 63, 90%) and the remaining 10% of the positive samples (*n* = 7) were recovered from the CSF. The distribution of IPD cases by epidemiological year and age group is summarized in Table 1.

### 3.1. Serotype Distribution

**Figure 1:**
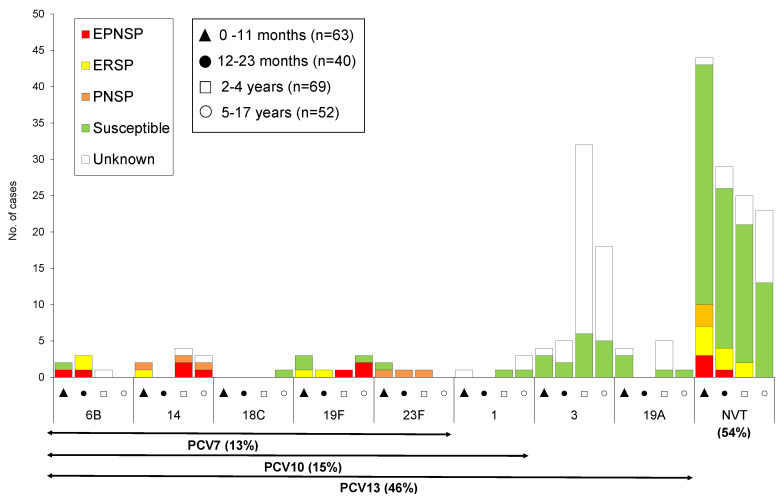
Number of samples representing serotypes present in conjugate vaccines causing invasive infections in Portugal (2015–2016 to 2017–2018). The number of samples representing each serotype in each of the age groups considered is indicated. Isolates presenting both erythromycin resistance and penicillin nonsusceptibility (EPNSP) are represented by red bars. Penicillin non-susceptible isolates (PNSP) are indicated by orange bars. Erythromycin-resistant isolates (ERSP) are indicated by yellow bars. Isolates susceptible to both penicillin and erythromycin are represented by green bars. Where pneumococci were detected exclusively by PCR or where the isolate was lost before antimicrobial susceptibility testing and for which susceptibility is unknown, are indicated by white bars. The serotypes included in each of the conjugate vaccines are indicated by the arrows. NVT—non-vaccine serotypes, i.e., serotypes not included in any of the currently available conjugate vaccines (PCV7, PCV10 and PCV13). The values indicated below the arrows are the proportion of each group in the overall cases (*n* = 223). Serotypes 4, 5, 7F, 9V, 18C included in PCV13, PCV10 or PCV7 were not detected in the study years.

Among the 223 isolates and patient samples, 30 different capsular types were detected (Figure 1). In 15 cases in which pneumococci were directly identified by PCR from a patient sample, it was not possible to unambiguously determine the serotype. In three samples, a group of related serotypes could be present in each of the samples—11A/11D (*n* = 1), 12A/12B/12F (*n* = 1) and 15B/C (*n* = 1). In 12 samples (5.4%) a serotype could not be identified by PCR but since our PCR reaction identified all serotypes present in PCV13 these samples were classified as NVT. Additionally, given the high frequency of spontaneous switching between serotypes 15B (*n* = 7) and 15C (*n* = 1) we decided to group isolates with these serotypes into a single group, 15B/C (*n* = 9, including the one patient sample in which the serotype was determined by PCR and no distinction between the two could be made). Given difficulties in unequivocally distinguish phenotypically isolates of serotypes 25A and 38, as well as serotype 29 and 35B, these were also grouped together into 25A/38 (*n* = 7) and 29/35B (*n* = 4).

**Figure 2:**
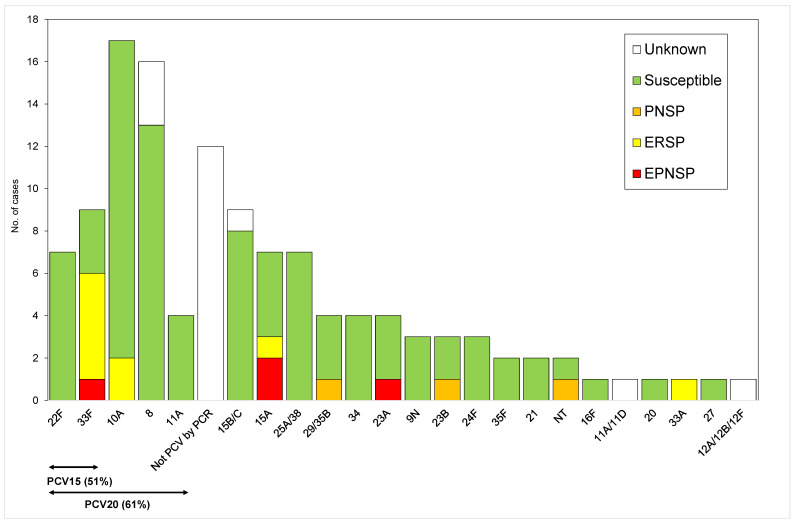
Number of samples representing serotypes not present in previously and currently available conjugate vaccines causing invasive infections in Portugal (2015–2016 to 2017–2018). See the legend of Figure 1. NT—Non-typeable. Some serotypes could not be unambiguously determined and are indicated as such (see text). Among the 15B/C IPD cases are eight in which isolates could be recovered—15B (*n* = 7) and 15C (*n* = 1)—and one detected by PCR. The proportion of isolates expressing serotypes represented in PCV15 and PCV20 is 51% and 61%, respectively. The potential coverage of PCV20 was calculated excluding the three samples in which the serotypes could not be unambiguously identified and that included PCV20 serotypes: 15B/C, 11A/D and 12A/B/F. Since our PCR serotyping schema does not detect serotype 10A it is possible that some of the samples for which a serotype could not be identified (“Not PCV by PCR”) represent this serotype which could further increase the potential coverage of PCV20.

Overall, the most frequent serotypes were serotype 3 (*n* = 59, 26.5%), followed by serotype 10A (*n* = 17, 7.6%), serotype 8 (*n* = 16, 7.2%) and serotype 19A (*n* = 10, 4.5%). PCV7 serotypes represented 12.6% of the cases (*n* = 28) of which only serotypes 14 (*n* = 9), 19F (*n* = 8), 6B (*n* = 6), 23F (*n* = 4) and 18C (*n* = 1) were detected (Figure 1 and Figure 2). Among the addPCV10 serotypes only serotype 1 was detected (*n* = 5, 2.2%) and only in the first two epidemiological years. Overall, a considerable proportion of IPD cases were still associated to the addPCV13 serotypes (*n* = 69, 30.9%). Among the NVTs the addPCV20 serotypes accounted for a large fraction of the isolates (*n* = 37), mostly due to serotypes 8 and 10A which were among the most frequent overall, while the addPCV15 serotypes were represented by fewer isolates (*n* = 16). Two isolates were non-typeable. Among the cases in which pneumococci were identified in the CSF the following serotypes were found: 8, 15B/C, 19A (*n* = 3 each); 3, 15A, 34, 22F, 19F (*n* = 2 each); and 6B, 9N, 10A, 12A/12B/12F, 21, 24, 25A/38, 27, 29/35B (*n* = 1 each).

Considering the serotypes of the previously and currently available conjugate vaccines, it was possible to detect significant differences when comparing the period in analysis with the previously reported period (2012–2015). While the proportion of PCV7 and addPCV10 serotypes decreased from 21.6% to 12.6% (*p* = 0.010) and from 15.9% to 2.2% (*p* < 0.001), respectively, the proportion of cases associated with addPCV13 increased from 20.3% to 30.9% (*p* = 0.010). An increase was also seen in the proportion of NVT cases, from 42.2% in 2012–2015 to 54.3% in 2015–2018 (*p* = 0.011). Moreover, when considering individual serotypes (*n* > 10 when considering both periods) in all age groups, we found significant differences in the proportion of some serotypes in pIPD, when comparing to the previous period (2012–2015). Significant increases were seen in the proportion of serotypes: 3 (from 13.8% to 26.5%, *p* = 0.001), 8 (from 2.2% to 7.2%, *p* = 0.013) and 33F (from 0.4% to 4%, *p* = 0.010), all significant after FDR correction. In contrast, significant decreases were detected in the proportion of pIPD of the PCV13 serotypes: 1 (from 9.9% to 2.2%, *p* = 0.007), 14 (from 9.9% to 4.0%, *p* = 0.017) and 7F (from 6.0% to 0%, *p* < 0.001), all significant after FDR correction. Non-significant increases were observed in the PCV serotype 19F (from 2.6% to 3.6%), and NVT serotypes 10A (from 4.3% to 7.6%) and 15A (from 1.3% to 3.1%). Non-significant decreases in the PCV serotypes 19A (from 5.6% to 4.5%), 23F (from 2.6% to 1.8%) and 6B (from 5.2% to 2.7%), as well as NVT serotypes 15B/C (from 5.2% to 4%) and 24F (from 4.3% to 2.7%), were also observed.

The serotype distribution among the different age groups is summarized in Table 2. Considering only serotypes with >5 isolates, we found that serotype 3 cases increased with age (*p* < 0.001, significant after FDR correction), whereas the NVT serotypes 15B/C and 33F cases decreased with age (*p* = 0.020 and *p* = 0.010, respectively, but neither significant after FDR correction). Overall, infections in younger children were more frequently associated with NVT serotypes (71.0% and 74.4% in the age groups 0–11 months and 11–23 months, respectively) (*p* < 0.001).

### 3.2. Antimicrobial Susceptibility

Antimicrobial susceptibility testing was performed on 151 available isolates (2 isolates were lost after serotyping) and is summarized in Figure 1 and Figure 2 and in Table 3. Overall, 21 isolates (13.9%) were nonsusceptible to penicillin (PNSP), of which all expressed low-level resistance. Considering the current CLSI breakpoints for parenteral penicillin, *n* = 6/24 (25%) CSF isolates would have been considered resistant and, assuming none of the other isolates would be associated with meningitis cases, none of those would have been considered non-susceptible. If we also apply the penicillin meningitis breakpoints to blood isolates, *n* = 13/114 (11%) would have been considered resistant. A blood isolate was considered non-susceptible to ceftriaxone and cefotaxime with the non-meningitis breakpoints (MICs of 2.0 µg/mL and 1.5 µg/mL, respectively). If using the meningitis breakpoints this isolate would have been considered resistant to ceftriaxone and cefotaxime and two additional blood isolates would have been considered non-susceptible. Resistance to erythromycin was found in 26 isolates (17.2%), of which the majority expressed the MLS_B_ phenotype (84.6%) and only 15.5% of the isolates expressed the M phenotype.

Comparing with the previous period, PNSP decreased significantly from 23.2% in 2012–2015 to 13.9% in 2015–2018 (*p* = 0.036) and erythromycin resistance showed a non-significant decrease from 22.7% in 2012–2015 to 17.2% in 2015–2018 (*p* = 0.220). Among the PCV13 serotypes, only the PCV7 serotypes 6B, 14, 19F and 23F were associated with antimicrobial non-susceptibility, while among NVT serotypes, serotypes 15A and 33F were the major contributors to antimicrobial resistance (Figure 1 and Figure 2). Among PNSP, serotype 14 was the most frequent (15.4%), while 3 serotypes accounted for more than half of erythromycin-resistant isolates: 33F (23.1%), 19F (19.2%) and 14 (15.4%).

## 4. Discussion

Although the number of cases identified by molecular methods remained almost stable from 2008–2012 and 2012–2015, their proportion of the overall pIPD cases increased [3]. In 2015–2018, the overall number of pIPD cases identified by molecular methods increased substantially (from *n* = 47 in 2012–2015 to *n* = 70) and this was also mirrored in an increase in the proportion of molecularly identified pIPD cases, from 20.2% to 30.1% (*p* = 0.007). This reflects mostly the increasing importance of complicated pneumonia in pIPD in Portugal in which pneumococci were frequently identified exclusively by molecular methods in pleural fluid and suggests that the incidence of pediatric complicated pneumonia in Portugal could be increasing, similarly to what was reported recently in Australia [21]. Serotype 3 has been shown to be a major cause of complicated pneumonia worldwide [11,12,21,22] and it was the most frequently detected serotype in pIPD in Portugal in 2015–2018 (Figure 1), predominantly detected in pleural fluid samples by molecular methods (*n* = 43/59, 72.8%).

A persistence of serotype 3 in pIPD is not universally reported, with some countries finding decreases in infections caused by this serotype following PCV13 vaccination [8,9,10]. However, the fact that many studies did not include cases detected by molecular methods could be a major reason behind these differences since it was reported by several groups that serotype 3 infections are more likely detected when using PCR rather than culture, particularly in cases of complicated pneumonia with pleural effusion [11,12,21,22,23].

Similar to the situation described here, other countries failed to detect decreases in serotype 3 IPD cases. A study in England and Wales found decreases in serotype 3 infections only in the older age groups (5–64 years and ≥65 years) but not in younger children (<5 years) up to 2014 [7] and more recent data from England point to an increase in serotype 3 IPD [24]. In France, a rebound in the incidence of serotype 3 IPD in children <2 years to pre-PCV13 levels was seen in recent years [25]. In Switzerland, after decreases since 2009, there was a rebound in serotype 3 IPD incidence in children <5 years in 2018–2019 [26]. In Spain, despite an overall reduction in IPD incidence between 2006 and 2014, the incidence of serotype 3 disease did not change in children <5 years, in a situation of intermediate vaccination coverage [27]. Additionally, in the same region of Spain, a study conducted between 2012 and 2016, allowed the identification of a significant number of vaccine failures associated with serotype 3, also the most frequent PCV13 serotype causing IPD in the region [22]. In agreement with what was found in Portugal in the current study, the authors found more serotype 3 IPD cases in older children (24–59 months) and the proportion of serotype 3 in age-appropriately vaccinated children was higher than in unvaccinated patients [22], similarly to the situation previously described in Portugal when analyzing complicated pneumonia cases between 2010 and 2015 [12]. National data from pIPD in Spain in 2019, 3-years after the introduction of PCV13 in the NIP, also found serotype 3 among the leading causes of pIPD, together with serotypes 8 and 24F [28]. In Germany, after the introduction of PCV13, and in a situation of high vaccination coverage, serotype 3 pneumonia with parapneumonic pleural effusion or empyema increased and a considerable number of vaccine failures were associated with this serotype [11]. All these results seem to support the lower effectiveness of PCV13 against serotype 3 complicated pneumonia, although unexplained geographical differences persist [29]. The reasons behind these differences could be related to different vaccination schedules, risk factors, natural trends, differences in sampling or transmission dynamics, as already suggested to account for other differences in serotype replacement [29,30]. Differences in the etiologic diagnosis methods could potentially further affect the results since the use of molecular methods to complement culture is essential to avoid underestimating this important serotype in the epidemiology of pIPD.

The reasons for the lower frequency of serotype 3 infections among children aged <2 years in our study are unclear, and we can only speculate. One of the possible reasons could be that older children with serotype 3 IPD were not vaccinated. In Portugal, PCV13 has been in the NIP since July 2015, and some of the older children (>2 years) in our study could, in fact, be unvaccinated. However, vaccination coverage in Portugal was estimated to be substantial (62%) even before PCV13 was in the NIP, so differences in the vaccination status between children <2 years and older children is unlikely to be the sole reason for the differences in the prevalence of serotype 3 infections in each group. It is possible that vaccine failures involving serotype 3 occur in vaccinated older children due to the waning of immunity to this serotype over time. One of the reasons for the lower effectiveness of PCV13 against serotype 3 was suggested to be the higher IgG levels necessary to protect against this serotype [31] suggesting that, in contrast to other serotypes, even modest antibody waning could lead to a significant lowering of protection. A study conducted in Massachusetts failed to detect this, with no association between the date of the last vaccine dose and time to IPD, but the number of serotype 3 infections was low, potentially not allowing a proper evaluation of the dynamics of this serotype [32].

The geographical differences in the importance of PCV13 serotypes are not exclusive of serotype 3. In the USA, significant reductions of IPD after 5 years of PCV13 were accompanied by major decreases in serotype 19A, but in Europe, this serotype continues to be a significant cause of disease, despite the use of PCV13 [10,13,24,25,28]. In Portugal, although a decrease was seen when comparing with the previous study period, serotype 19A still ranked fourth in pIPD. Contrarily to the situation of serotype 14, which is associated with antimicrobial non-susceptibility potentially contributing to its persistence, most isolates expressing serotype 19A are now fully susceptible to all antimicrobials tested (Figure 1), ruling out resistance as the reason for the persistence of this serotype. Serotype 19F infections increased non-significantly when compared with the previous period, which is worrisome and should be closely monitored, since this serotype was shown to persist, albeit at low levels, as a cause of IPD in most settings despite being included in PCV7, which has been used for nearly two decades [33]. Similar to serotype 14, the association of serotype 19F with antimicrobial non-susceptibility raises the possibility that antimicrobial use could contribute to its persistence.

The increase in NVTs in pIPD seen here, although reported to be more pronounced in the adult population [5,33], was also documented in children in many other countries [7,10,24,25,34]. In agreement with what was reported in this study, in some studies, it was possible to detect an increase in NVTs in particular age groups, as in children aged <5 years [7] or <2 years [10]. The increase in serotype 33F when compared with the previous period could be explained by its association with IPD in younger children and antimicrobial resistance, reinforcing the need to closely monitor this emergent serotype. Another important emergent NVT serotype in pIPD is serotype 8. In Portugal, this serotype was already identified as an important cause of IPD in adults, with a sustained increase in this population since 2008 [4,5,35], being the second most important cause of adult IPD in 2012–2014 [5] and rising to the most frequent in 2015–2018 [35]. Whether the increase in pIPD will be sustained in the future is still uncertain but its dynamics in the adult population suggests that it may become an important cause of overall IPD. Serotype 8 was also identified as an important cause of IPD in other countries where non-PCV13 serotypes increased as causes of pIPD [10,24,25], reinforcing the need to closely monitor this emergent serotype previously shown to have an enhanced invasive disease potential [36].

Overall, after PCV13 implementation, serotype replacement was not restricted to a limited number of serotypes and instead there is now a more diverse group of serotypes associated with IPD, which also show temporal and geographic differences. In Europe, the leading causes of NVT disease were found to be serotypes 12F, 10A, 24F, 22F and 15C; while in North America serotypes 22F, 33F, 15B, 38 and 35B were important [13]. The data presented here indicate that these geographic differences may be attenuating since several of the North American serotypes were also important causes of pIPD in Portugal.

The two expanded valency vaccines currently being approved would offer different potential gains in terms of pIPD prevention, but neither would greatly enhance PCV13 coverage. While PCV15 would cover an additional 7.2% of pIPD cases, PCV20 would more than double that to cover an additional 16.6% of cases (the potential coverage of PCV20 was calculated excluding the three samples in which the serotypes could not be unambiguously identified and that included PCV20 serotypes: 15B/C, 11A/D and 12A/B/F—Figure 2).

Our study has several limitations. The first is our inability to calculate IPD incidence because we did not receive information regarding cases for which isolates or samples were not submitted for characterization. However, we believe we are not missing a large number of cases because in our previous population-based studies, missing samples represented from 8 to 12% of yearly cases [3]. The stability of our surveillance network, the active nature of the surveillance and the involvement of both pediatric and microbiology departments of many hospitals covering the entire country, further substantiates the identification of most cases. Therefore, the small decrease in the total number of isolates and pneumococcal positive samples received in this period (*n* = 223) relative to the previous period (*n* = 232) may be interpreted as evidence of continued stability of the incidence of pIPD in Portugal. Regarding the serotype distribution of missing samples, we cannot guarantee that their serotypes would follow the serotype distribution of available samples, but the expected small proportion of cases with unknown serotype information ensures that our results would not be strongly affected. Lastly, our study was not designed to obtain information allowing us to assess the severity of infections caused by the different serotypes (e.g., hospitalization, ICU admission, 30-day mortality) and we also did not have comprehensive information on the vaccination status of the cases, which were essential to identify vaccine failures.

## 5. Conclusions

In Portugal, after a period of a substantial decline in pIPD, the incidence seems to have stabilized and PCV13 serotypes remain an important cause of disease, even in a situation of a mature vaccination program [37] with 3-years of universal PCV13 vaccination. Serotype 3 accounted for over a quarter of all cases, increasing relative to the previous period of non-universal vaccination. Moreover, the use of molecular methods was instrumental for the identification of this serotype in pleural fluid, which represented 31% of all samples, reinforcing the need to use molecular methods to fully understand the dynamics of pIPD. The increase in NVTs is worrisome, especially serotypes 33F, due to its association with antimicrobial non-susceptibility, and 8, due to its increasing importance as an emergent serotype in both pIPD and adult IPD in Portugal. These observations reinforce the need to continue surveillance studies to better understand the benefits of current vaccination strategies and to evaluate the potential benefits of future vaccines.

## Figures and Tables

**Table 1:** Cases of invasive pneumococcal disease from patients <18 years, Portugal, July 2015–June 2018 (*n* = 223).

Epidemiological Years ^1^	Cases by Age Group				
	0–11 Months	12–23 Months	2–4 Years	5–17 Years	Total
2015–2016	16	16	18	17	67
2016–2017	23	6	25	19	73
2017–2018	23	17	27	16	83
Total	62	39	69	52	223

^1^ From 1 July to 30 June of the following year.

**Table 2:** Serotype distribution of *Streptococcus pneumoniae* isolates responsible for invasive disease in patients < 18 years, Portugal, July 2015–June 2018, among the different age groups (for serotypes with *n* > 5).

Serotype	0–11 Months *n* (%)	12–23 Months *n* (%)	2–4 Years *n* (%)	5–17 Years *n* (%)	CA ^1^
1	1 (1.6%)	0 (0%)	1 (1.4%)	3 (5.8%)	0.16
3	4 (6.5%)	5 (12.8%)	32 (45.7%)	18 (34.6%)	**<0.001**
8	7 (11.3%)	1 (2.6%)	4 (5.7%)	4 (7.7%)	0.50
14	2 (3.2%)	0 (0%)	4 (5.7%)	3 (5.8%)	0.29
10A	6 (9.7%)	5 (12.8%)	4 (5.7%)	2 (3.8%)	0.15
15A	3 (4.8%)	2 (5.1%)	1 (1.4%)	1 (1.9%)	0.24
15B/C ^2^	5 (8.1%)	3 (7.7%)	0 (0%)	1 (1.9%)	0.02
19A	4 (6.5%)	0 (0%)	5 (7.1%)	1 (1.9%)	0.57
19F	3 (4.8%)	1 (2.6%)	1 (1.4%)	3 (5.8%)	0.99
22F	3 (4.8%)	0 (0%)	4 (5.7%)	0 (0%)	0.40
25A/38	1 (1.6%)	3 (7.7%)	3 (4.3%)	0 (0%)	0.62
33F	5 (8.1%)	3 (7.7%)	1 (1.4%)	0 (0%)	0.01
6B	2 (3.2%)	3 (7.7%)	1 (1.4%)	0 (0%)	0.14

^1^ CA: Cochran–Armitage test for trend. In bold are values significant after FDR correction. ^2^ The actual number of isolates of each serotype was 15B, *n* = 7 and 15C, *n* = 1. In one case the serotype was determined directly from the patient sample and no distinction between these serotypes was possible.

**Table 3:** Antimicrobial resistance of *Streptococcus pneumoniae* isolates responsible for invasive disease in patients <18 years, Portugal, July 2015–June 2018 (*n* = 151).

Antibiotic ^1^	Number of Resistant Isolates (%) ^2^			
	0–11 Months (*n* = 58)	12–23 Months (*n* = 33)	2–4 Years (*n* = 34)	5–17 Years (*n* = 26)
PEN ^3^	9 (15.5)	3 (9.1)	5 (14.7)	4 (15.4)
MIC_90_^4^	0.125	0.023	0.25	0.5
MIC_50_^4^	0.012	0.012	0.012	0.008
CRO ^3^	0 (0)	0 (0)	1 (2.9)	0 (0)
MIC_90_^4^	0.047	0.032	0.25	0.38
MIC_50_^4^	0.012	0.012	0.012	0.012
CTX ^3^	0 (0)	0 (0)	1 (2.9)	0 (0)
MIC_90_^4^	0.047	0.032	0.38	0.38
MIC_50_^4^	0.016	0.016	0.016	0.012
ERY	10 (17.2)	8 (24.2)	5 (14.7)	3 (11.5)
CLI	9 (15.5)	6 (18.2)	4 (11.8)	3 (11.5)
CHL	0 (0)	1 (3.0%)	0 (0)	0 (0)
SXT	5 (8.6)	5 (15.2)	4 (11.8)	2 (7.7)
TET	8 (13.8)	5 (15.2)	4 (11.8)	1 (3.8)
VAN	0 (0)	0 (0)	0 (0)	0 (0)
LVX	0 (0)	0 (0)	0 (0)	0 (0)
LZD	0 (0)	0 (0)	0 (0)	0 (0)

^1^ CHL: chloramphenicol; CLI: clindamycin; CRO: ceftriaxone; CTX: cefotaxime; ERY: erythromycin; LVX: levofloxacin; LZD: linezolid; PEN: penicillin; SXT: trimethoprim–sulfamethoxazole; TET: tetracycline; VAN: vancomycin. ^2^ Unless otherwise specified. ^3^ Number and percentage of non-susceptible isolates are indicated. ^4^ MIC—minimal inhibitory concentration inhibiting the growth of 50% (MIC_50_) or 90% (MIC_90_) of the isolates. Values shown are in µg/mL.

## Data Availability

All the data in this study is available in the figures and tables of the paper.
